# Acipimox Administration With Exercise Induces a Co-feedback Action of the GH, PP, and PYY on Ghrelin Associated With a Reduction of Peripheral Lipolysis in Bulimic and Healthy-Weight Czech Women: A Randomized Study

**DOI:** 10.3389/fendo.2019.00108

**Published:** 2019-03-12

**Authors:** Kvido Smitka, Jara Nedvidkova, Karel Vondra, Martin Hill, Hana Papezova, Vojtech Hainer

**Affiliations:** ^1^Laboratory of Clinical and Experimental Neuroendocrinology, Institute of Endocrinology, Prague, Czechia; ^2^First Faculty of Medicine, Institute of Physiology, Charles University, Prague, Czechia; ^3^First Faculty of Medicine, Institute of Pathological Physiology, Charles University, Prague, Czechia; ^4^Clinical Department, Institute of Endocrinology, Prague, Czechia; ^5^Steroid Hormone and Proteofactors Department, Institute of Endocrinology, Prague, Czechia; ^6^First Faculty of Medicine, Eating Disorder Center, Psychiatric Clinic, Charles University and General University Hospital, Prague, Czechia; ^7^Obesity Management Center, Institute of Endocrinology, Prague, Czechia

**Keywords:** eating disorders, exercise, growth hormone, ghrelin, microdialysis, olbetam, pancreatic polypeptide family, human adipose tissue

## Abstract

**Objective:** Anti-lipolytic drugs and exercise are enhancers of growth hormone (GH) secretion. Decreased circulating free fatty acids (FFA) have been proposed to exert ghrelin-GH feedback loop after administration of an anti-lipolytic longer-acting analog of nicotinic acid, Acipimox (OLB, 5-Methylpyrazine-2-carboxylic acid 4-oxide, molecular weight of 154.1 Da). OLB administration strongly suppresses plasma FFA during exercise. Neuroendocrine perturbations of the adipose tissue (AT), gut, and brain peptides may be involved in the etiopathogenesis of eating disorders including bulimia nervosa (BN) and anorexia nervosa. BN is characterized by binge eating, self-induced vomiting or excessive exercise.

**Approach:** To test the hypothesis that treatment with OLB together with exercise vs. exercise alone would induce feedback action of GH, pancreatic polypeptide (PP), peptide tyrosine tyrosine (PYY), and leptin on ghrelin in Czech women with BN and in healthy-weight Czech women (HW). The lipolysis rate (as glycerol release) in subcutaneous abdominal AT was assessed with microdialysis. At an academic medical center, 12 BN and 12 HW (the control group) were randomized to OLB 500 mg 1 h before a single exercise bout (45 min, 2 W/kg of lean body mass [LBM]) once a week vs. identical placebo over a total of 2 weeks. Blood plasma concentrations of GH, PP, PYY, leptin, ghrelin, FFA, glycerol, and concentrations of AT interstitial glycerol were estimated during the test by RIA utilizing ^125^I-labeled tracer, the electrochemiluminescence technique (ECLIA) or colorimetric kits.

**Results:** OLB administration together with short-term exercise significantly increased plasma GH (*P* < 0.0001), PP (*P* < 0.0001), PYY, and leptin concentrations and significantly decreased plasma ghrelin (*P* < 0.01) concentrations in both groups, whereas short-term exercise with placebo resulted in plasma ghrelin (*P* < 0.05) decrease exclusively in BN. OLB administration together with short-term exercise significantly lowered local subcutaneous abdominal AT interstitial glycerol (*P* < 0.0001) to a greater extent in BN.

**Conclusion:** OLB-induced suppression of plasma ghrelin concentrations together with short-term exercise and after the post-exercise recovering phase suggests a potential negative co-feedback of GH, PP, PYY, and leptin on ghrelin secretion to a greater extent in BN. Simultaneously, the exercise-induced elevation in AT interstitial glycerol leading to a higher inhibition of peripheral lipolysis by OLB in BN.

**Clinical Trial Registration:** www.ClinicalTrials.gov, identifier NCT03338387

## Introduction

Bulimia nervosa (BN) and anorexia nervosa (AN) are serious eating disorders that can persist for years and contribute to increased mortality (1). AN being a psychiatric disorder with the highest mortality (2) is manifested by chronic self-induced starvation, amenorrhea and severe weight loss, mainly at expense of adipose tissue (AT), and refusal to regain and maintain a minimal body weight, while for BN recurrent episodes of over-eating followed by inadequate substitute behavior including spontaneously induced vomiting, laxatives, diuretics and excessive use of enemas, fasting or excessive exercise, and dysregulation of several endogenous neuroimmunoendocrine axes are typical (3, 4).

**Figure 1:**
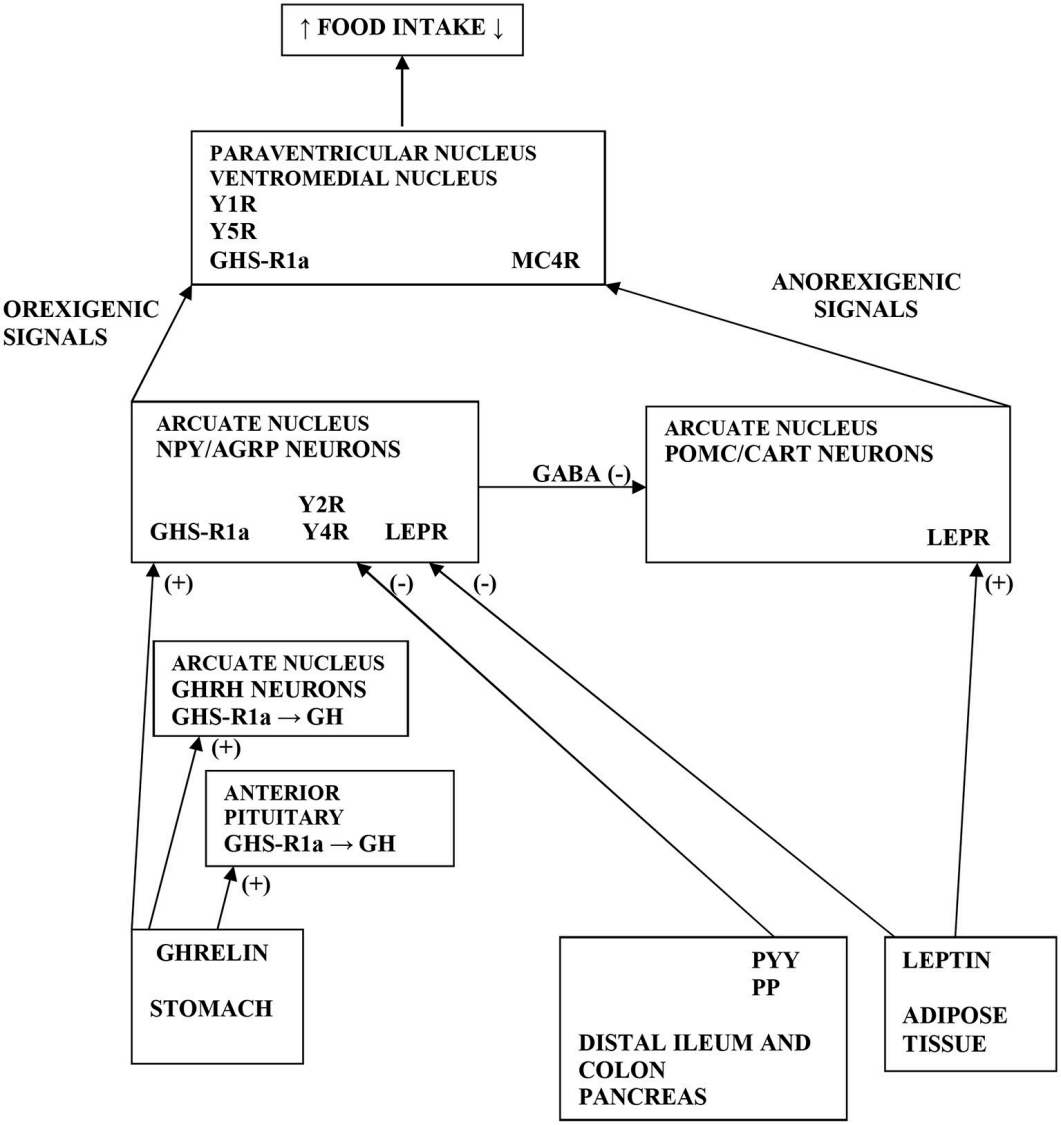
Scheme demonstrating the various pathways of food intake regulations. Ghrelin is produced by stomach and stimulates GH release by acting GHS-R1a in the anterior pituitary and on GHRH neurons in the ARC of hypothalamus. Further, ghrelin stimulates the NPY/AGRP neurons *via* GHS-R1a in the ARC. GHS-R1a in the hypothalamus is also expressed in PVN and VMH. Moreover, ghrelin *via* GHS-R1a increases food intake by releasing of GABA inhibitory input from NPY/AGRP neurons to POMC/CART neurons in the ARC. PYY and PP are secreted from distal ileum, colon and pancreas to produce an inhibitory effect on NPY/AGRP neurons *via* Y2R and Y4R in the ARC. Leptin is secreted from AT to inhibit the NPY/AGRP neurons and stimulate the POMC/CART neurons *via* LEPR in the ARC. Elevated activity of POMC neurons increases alpha-MSH release in the PVN, which acts on MC4R to inhibit food intake. Orexigenic signals from the NPY neurons act on Y1R and Y5R in the PVN, whereas AGRP antagonizes MC4R. NPY, neuropeptide tyrosine; GH, growth hormone; ARC, arcuate nucleus; PVN, paraventricular nucleus; VMH, ventromedial nucleus; AT, adipose tissue; LEPR, leptin receptor; GHS-R1a, growth hormone secretagogue receptor type 1a; GHRH, growth hormone releasing hormone; POMC, pro-opiomelanocortin; CART, cocaine and amphetamine related transcript; GABA, gamma-aminobutyric acid; AGRP, agouti-related protein; MC4R, melanocortin 4 receptor; alpha-MSH, alpha-melanocyte-stimulating hormone; Y1, 2, 4, 5R, Y receptors; PP, pancreatic polypeptide; PYY, peptide tyrosine tyrosine; (+) = the stimulatory effect; (-) = the inhibitory effect.

BN, a serious mental illness, is characterized by AT-gut-brain axis dysfunction leading to disruption of appetite-stimulating (orexigenic) and appetite-suppressing (anorexigenic) peptides. In BN patients, orexigenic neuropeptides (ghrelin and neuropeptide tyrosine [NPY]) are up-regulated facilitating binge eating behavior; however, anorexigenic peptides (peptide tyrosine tyrosine [PYY], pancreatic polypeptide [PP], and leptin) are down-regulated underlying post-binge eating behavior (5–8) (Figure 1). These “mixed” signals may initiate or exacerbate episodes of binge-purging and post-binge eating cycles in BN (4). In our previous studies, we documented different responses of growth hormone (GH), ghrelin, NPY, and ACTH to treatment with the anti-hyperlipidemic drug Acipimox (OLB) in BN, and we confirmed that OLB with exercise indicates GH inhibition of ghrelin (4, 9–11). Thus, OLB administration has been shown as an enhancer of GH, ACTH, and leptin release. Indeed, OLB and its analogs may be used in the treatment of eating disorders for regulation of food intake, glucose disposal, FFA disarray as well as GH, ACTH, and leptin secretagogue activity. Therefore, OLB administration could maintain normal glycemia and survival in response to negative energy balance such as exercise or fasting in AN and BN patients.

In fact, PP, PYY, and NPY are members of a PP family that shares strong tertiary structural homology called the “PP-fold” which is essential for the function and maintaining bioactivities of all members (12). The PP family is one of the major systems modifying the anti-stress response, emotionality, anxiety- and depression-like behavior, and hormonal dysbalance relevant to AN and BN (13).

The release of NPY is one of the mechanisms by which ghrelin antagonizes leptin action and stimulates appetite through ghrelin receptors on NPY neurons in the hypothalamic arcuate nucleus (ARC) (14) (Figure 1). Recently, a novel peripheral site for NPY biosynthesis was found in human adipocytes where NPY stimulates proliferation of primary preadipocytes and participates in adipogenesis and liporemodeling in subcutaneous (sc) and visceral AT (15, 16). Ghrelin promotes PP synthesis and thus the powerful stimulating action of ghrelin on PP secretion was shown in humans (17). Ghrelin, PYY and GH may interact with each other. In bulimic women, a negative correlation was reported between meal induced PYY elevation and ghrelin suppression, confirming a negative interaction of PYY with ghrelin (6). Furthermore, GH levels also peak during the adolescent growth spurt implying that elevated GH levels cause a lowering of PYY levels, which facilitates increased food intake (18). Thus, the inability to down-regulate PYY levels during this crucial growth period might be the reason for the increased incidence of the development of AN (19). Patients with BN exhibited reduced levels of PP postprandially contributing to post-binge eating behavior, while administration of PP could ameliorate the binge eating aspects of BN (7).

The nicotinic acid family of receptors (recently classified as hydroxy-carboxylic acid [HCA] receptors) respond to the lipid-lowering agents such as nicotinic acid, OLB, acifran, phenolic acids, methyl fumarate, 3-hydroxy-butyrate, and 2-hydroxy-propionate (lactate) (20–27). The identification of 3-hydroxy-butyrate as an endogenous ligand for HCA2 receptor leads us to suggest that HCA2 receptors might play a critical role in maintaining of AT energy stores in survival of starvation in AN and BN, i.e., to conserve glucose in periods of fasting or excessive exercise (25). OLB may act *via* HCA2 receptor related and non-related mechanisms. However, the precise cellular mechanism of action of a niacin-like drug OLB by which it exerts its main effect, i.e., suppression of lipolysis from AT, is partly clarified. It is surmised that activation of G_i_-type G protein-coupled receptors formerly known as GPR109A on the membrane of adipocytes result in the subsequent suppression of cAMP (28, 29). Furthermore, HCA2 receptor is found in AT, stomach, intestinal epithelium, pancreatic islets, hypothalamic neurons, and the brain (30, 31). Nicotinic acid is synthesized from tryptophan and its deficiency results in pellagra, which is seen as a secondary complication among patients suffering from AN and BN (32).

Key hormones controlling adipocyte function are catecholamines such as norepinephrine (NE) and epinephrine (E). Catecholamines stimulate or inhibit lipolysis depending on the presence of tissue adrenoceptors involved in their effect. Stimulation of lipolytic beta adrenergic G_s_-type G protein-coupled receptors increases production of cAMP, whereas stimulation of anti-lipolytic alpha_2_-adrenergic G_i_-type G protein-coupled receptors decreases cAMP production. In humans, the increase of lipolysis in sc abdominal AT during physical exercise results from the interplay of stimulatory and inhibitory actions of catecholamines (33). As previously found by our group, the condition of malnourished, and underweight anorexic patients is associated with an *in vivo* increased basal sympathetic nervous system (SNS) activity and AT lipolysis and also exercise-induced SNS activity, particularly with respect to sc abdominal AT dialysate NE concentrations (34).

The primary purpose of this placebo-controlled study was to test the hypothesis whether an increase or suppression in plasma ghrelin concentrations during short-term exercise alone or with anti-lipolytic OLB administration is exerted by a feedback action of appetite-regulating hormones in Czech women with BN and in healthy-weight Czech women (HW). The secondary purpose of this randomized study was to examine the responses of local sc abdominal AT dialysate extracellular glycerol concentrations to short-term exercise together with OLB or placebo administration in BN and HW evaluated by an *in vivo* microdialysis technique.

## Subjects and Methods

The study was performed in accordance with the Declaration of Helsinki and was approved by the Ethics Committee of the Institute of Endocrinology in Prague. Each participant signed an informed consent form before entering the study. The study is registered at ClinicalTrials.gov; Identifier: NCT03338387.

### Subjects

Twelve Czech women with purging subtype of BN (means ± S.E.M., age: 23.45 ± 1.2 years; body mass index [BMI]: 21.85 ± 0.60 kg/m^2^; body fat content determination [% BF]: 22.7 ± 0.75) and 12 healthy-weight Czech women (HW) (age: 22.63 ± 1.49 years; BMI: 20.76 ± 0.88 kg/m^2^; % BF: 21.5 ± 2.46) were recruited for this study (Table 1). Healthy controls did not have a history of ischemic heart disease, restricting or purging subtype of AN, purging or non-purging subtype of BN or any subtypes of night binge-eating disorder or other psychiatric disorders. Adult HW were recruited for the study, comprising high school students, university students, clercks, and office workers. All patients fulfilled DSM-IV-TR diagnoses for purging subtype of BN (3). The questionnaires EDE-Q, MINI, and EDI-2 were used to differentiate eating disorder subtypes and exclude comorbidity cases and drop-outs. During a clinical examination, 3 bulimic women had amenorrhea, 4 bulimic women had oligomenorrhea and 5 bulimic women were in the early follicular phase of the menstrual cycle. In the bulimic patients, the average frequency of binge-eating and binge-purging episodes was 2.7 times per day and the average duration of their eating disorder was 7 years and 10 months. They were admitted and investigated during their first week of hospitalization at the Eating Disorder Center. Inclusion and patient and healthy control selection: The controls were healthy-weight Czech women aged between 18 and 30 years and with a body mass index (BMI) between 19 and 23 kg/m^2^; the patients were Czech women with the diagnosis of purging subtype of BN. However, diabetic, pregnant or lactating women, patients with any severe active infection, patients with clinically significant chronic disease involving the cardiovascular, hematopoietic, hepatic, hepatogastroenteric or urinary systems, patients with abnormal blood tests with significant hyperlipidemia, and patients with hypo- or hyperthyroidism, impaired mental capacity or markedly abnormal psychiatric evaluation were excluded. Among all of the bulimic patients (20) and HW (18), 12 Czech women with purging subtype of BN and 12 healthy-weight Czech women were acceptable during the inclusion procedure (the recruitment phase in the Institute of Endocrinology) for being randomized, i.e., 5 bulimic women were excluded, 1 bulimic woman did not meet the inclusion criteria, 2 bulimic women declined to participate in the study, 3 HW did not meet the inclusion criteria and 3 HW declined to participate in this study. The administration of OLB was well-tolerated. Common adverse effects included skin flushing in all subjects receiving OLB.

**Table 1:** Baseline characteristics including anthropometric measurements and circulatory response of the study subjects during rest and the exercise alone or together with Acipimox treatment.

**Age (years)**	**Week + placebo**		**Week + OLB**
HW group (*n =* 12)	22.63 ± 1.49 NS		22.63 ± 1.49 NS
BN group (*n =* 12)	23.45 ± 1.2 NS		23.45 ± 1.2 NS
**BMI (kg/m**^**2**^ **)**			
HW group (*n =* 12)	20.76 ± 0.88 NS		21.6 ± 0.75 NS
BN group (*n =* 12)	21.85 ± 0.6 NS		22.36 ± 0.5 NS
**% BF**			
HW group (*n =* 12)	21.5 ± 2.46 NS		22.4 ± 2.6 NS
BN group (*n =* 12)	22.7 ± 0.75 NS		23.1 ± 0.8 NS
**Heart rate (bpm)**	**Rest**	**45-min Exercise + placebo 500 mg p.o**.	**45-min Exercise + OLB 500 mg p.o**.
HW group (*n =* 12)	72 ± 2.5	135 ± 8.9^***^	148 ± 9.3^***+^
BN group (*n =* 12)	55 ± 4.5^$^	145 ± 7.7^***^	155 ± 5.7^***^^+^
**Systolic blood pressure (mmHg)**	**Rest**	**45-min Exercise + placebo 500 mg p.o**.	**45-min Exercise + OLB 500 mg p.o**.
HW group (*n =* 12)	121 ± 4.7	147 ± 2.5^*^	150 ± 5.5^*^
BN group (*n =* 12)	90 ± 3.5^$^	135 ± 3.8^*^^$^	140 ± 7.2^*^
**Diastolic blood pressure (mmHg)**	**Rest**	**45-min Exercise + placebo 500 mg p.o**.	**45-min Exercise + OLB 500 mg p.o**.
HW group (*n =* 12)	72 ± 2.7	85 ± 2.7^*^	80 ± 3.5^*^
BN group (*n =* 12)	54 ± 3.5^$^	75 ± 3.2^*^	73 ± 2.5^*^

Values are means ± S.E.M. HW = healthy-weight Czech women (n = 12); BN = Czech women with bulimia nervosa (n = 12); BMI, body mass index; % BF, body fat content determination; bpm, beats per minute, NS, not significant; n = the number of subjects are in brackets, p.o., per os.

* P < 0.05,

*** P < 0.001 vs. resting (baseline) values,

$ P < 0.05 vs. HW,

+ *P < 0.05 exercise together with Acipimox (OLB) administration vs. exercise alone, 45 min*.

*Exercise results (from 120 to 165 min after the start of the experiment) are maximal values attained during the investigation (45 min, 2 W/kg of lean body mass [LBM])*.

### Experimental Protocol and Blood Sampling

**Figure 2:**
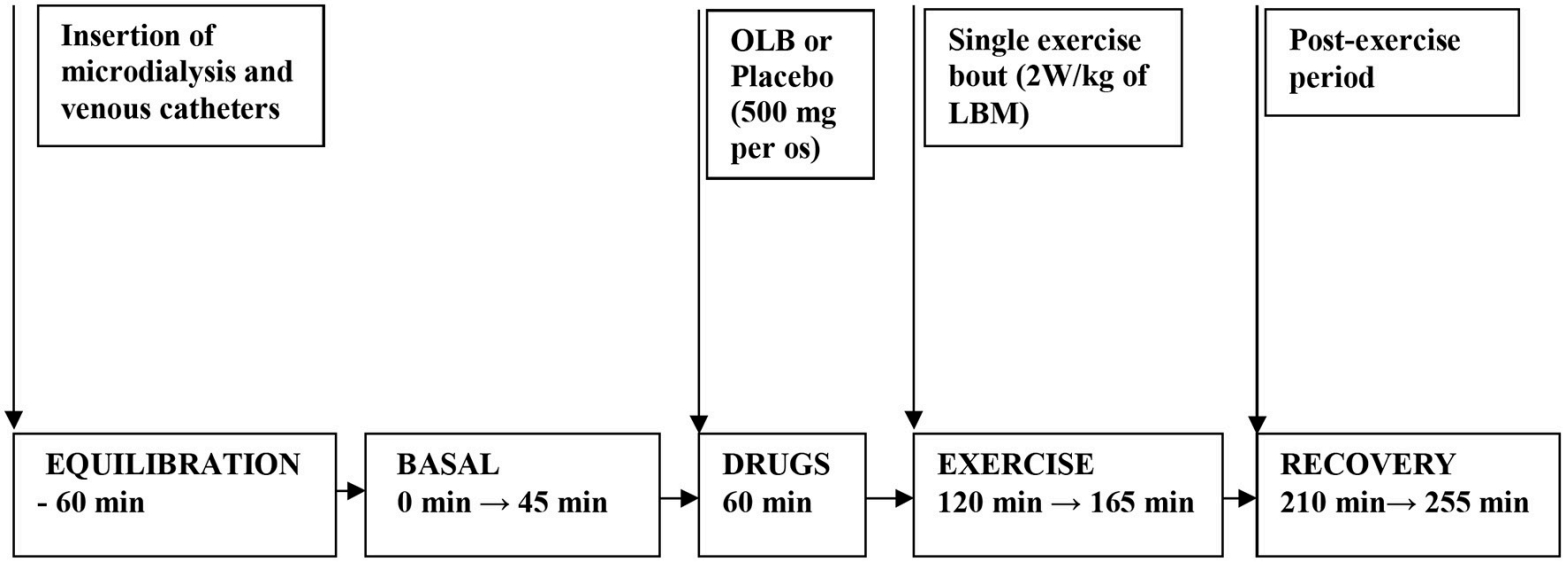
Flow chart depicting the experimental timeline when drugs and exercise were administered. Microdialysis and venous catheters were inserted at least 60 min before microdialysate and blood sampling. After 60 min of equilibration, basal microdialysate samples were collected in 45 min interval. OLB or placebo were administered orally 60 min before a single exercise bout. Then the subjects performed 45 min a single exercise bout (2 W/kg of LBM) followed by 90 min of recovery period. Microdialysate samples were collected 45 min during exercise and at last 45 min interval before ending the 90 min post-exercise recovering period. Blood samples were collected at 45 min of the investigation (basal) and at 165 min of the investigation (immediately after exercise) and at 255 min of the investigation (90 min after post-exercise recovering period). OLB, Olbetam; LBM, lean body mass.

Healthy volunteers and bulimic women were admitted to the Institute of Endocrinology at 7:00 AM. Screening tests included acute care panel and thyroid function tests. Patients and healthy women underwent measurements of height and weight, BMI, body fat content determination, electrocardiogram (ECG), and anthropometric measurements. Bioimpedance and anthropometric parameters were measured by TANITA apparatus (Tokyo, Japan). The placebo was matched to the study anti-hyperlipidemic drug OLB, and contained microcrystalline cellulose, identical in appearance but without the active ingredient. Each woman was assigned a serial number and received four capsules in two bottles. All subjects were randomized according to a computer generated randomization list to receive either placebo or OLB capsules each week (two 250 mg capsules of the acute-bolus OLB therapy or placebo; 500 mg in total−5-Methylpyrazine-2-carboxylic acid 4-oxide, molecular weight of 154.1 Da, Olbetam capsules, Farmitalia Carlo Erba, Milan, Italy) 1 h before a single exercise bout, once a week over a total of 2 weeks. A low- to moderate-intensity exercise bout on an electromagnetically braked bicycle ergometer (Cateye EC 1600, Japan) was performed for 45 min at power output 2 W/kg of lean body mass (LBM), and the average pedal speed was 60 rpm, intended to be below the aerobic-anaerobic threshold. Blood pressure and heart rate were monitored continuously using a 12-lead electrocardiogram (Eagle 3000 cardiomonitor, Marquette, Milwaukee, WI, U.S.A.) and hemodynamic parameters were measured every 5 min during the 45 min exercise bout (Table 1). At 8:00 AM, after overnight fasting, a venous catheter was inserted into the antecubital vein (Figure 2). A blood sample was collected at the beginning (baseline), after the 45 min exercise (at 165 min after the start of the experiment), and after a 90 min post-exercise recovering phase (at 255 min after the start of the experiment) to estimate plasma GH, ghrelin, PP, NPY, PYY, leptin, FFA, glycerol, insulin, and blood glucose levels. Approximately, 40 mL blood samples were collected into chilled tubes containing Na_2_ EDTA and antilysin to be used in the study. Blood plasma was separated immediately by centrifugation at 4°C and stored at −80°C until used.

### Characterization of Peripheral Lipolysis by Microdialysate Sampling and Glycerol Recovery

A CMA-60 microdialysis probe (CMA Microdialysis AB, Solna, Sweden) with membrane length 3 cm and molecular weight cut-off 20 kDa was inserted sc under sterile conditions (8–10 cm left of the umbilicus) after local anesthesia with 1% lidocaine. After insertion of the CMA-60 catheter, perfusion with sterile Ringer solution was started at a flow rate of 2 μL/min using a CMA 107 microdialysis pump (CMA Microdialysis AB, Solna, Sweden) (Figure 2). Microdialysate samples were collected 45 min at the beginning (baseline), 45 min during the exercise (at 165 min after the start of the experiment) and at the last 45 min interval before ending the 90 min post-exercise recovering phase (at 255 min after the start of the experiment) to estimate AT dialysate extracellular glycerol. The microdialysate volumes of samples measured at 45-min intervals were 96 ± 5 μL. Microvials were placed on ice immediately after the collection, and stored at −80°C until analysis. Before starting microdialysis perfusion, the relative glycerol recovery (RGR) was calculated *in vitro* at a temperature of 37°C maintained by a digital block heater to simulate body temperature. Different perfusion rates (0.1, 0.3, 0.5, 1.0, 2.0, 5.0 μL/min) were tested to investigate a possible relative recovery vs. perfusion rate dependency. At each rate, RGR was calculated from 20 samples collected in perfusion rate-dependent intervals according to the formula: RGR (%) = (glycerol concentration in dialysate/glycerol concentration in standard solution) × 100. In the present study, *in vitro* RGR calculation result was 86 ± 7% (*n* = 20) for the CMA-60 catheter (20 kDa cut-off, 3 cm membrane length, CMA Microdialysis AB, Solna, Sweden) at the perfusion rate of 2 μL/min. The flow rate of 2 μL/min was chosen for *in vivo* microdialysis and experiment duration.

### Hormonal and Bioanalytical Assays

GH in plasma (50 μL) [as the 22 kDa monomeric GH form and non-22 kDa isoforms (dimers and GH bound to plasma proteins)] was measured by a commercial RIA kit utilizing ^125^I-labeled tracer (Immunotech, Prague, Czech Republic). Intra- and inter-assay variability was 2 and 15%, respectively; sensitivity was 0.1 μIU/mL. Plasma ghrelin (200 μL) was measured by a commercial RIA kit utilizing ^125^I-labeled tracer (Linco Research, St. Charles, Missouri, U.S.A.). Sensitivity and the intra- and inter-assay coefficients of variation (CV) were 93 pg/mL, 14.7 and 10%, respectively. PP in plasma (100 uL) was measured by a commercial RIA kit utilizing ^125^I-labeled tracer (DRG Instruments GmbH, Marburg, Germany). Sensitivity, inter-assay CV and intra-assay CV were 3 pmol/L, 1.9 and 3.4%, respectively. NPY in plasma (200 μL) was determined by a commercial RIA kit utilizing ^125^I-labeled tracer (Linco Research Inc., St. Charles, Missouri, U.S.A.). The intra- and inter-assay variability for plasma NPY was 6.0 and 9.6%, respectively; sensitivity was 3 pmol/L. PYY in plasma (100 μL) was measured by a commercial RIA kit utilizing ^125^I-labeled tracer (Linco Research Inc., St. Charles, Missouri, U.S.A.). The intra- and inter-assay variability for plasma PYY was 3.9 and 8.7%, respectively; sensitivity was 10 pg/mL. Leptin in plasma (100 μL) was measured by a commercial RIA kit utilizing ^125^I-labeled tracer (Linco Research Inc., St. Charles, Missouri, U.S.A.). Sensitivity, inter-assay and intra-assay variability were 0.05 ng/mL, 9 and 6%, respectively. Insulin in plasma (5 μL) and blood glucose in plasma (5 μL) were estimated using an electrochemiluminescence method (ECLIA) or colorimetric technique in the Cobas Integra 400 plus system (Roche Diagnostics, GmbH, Mannheim, Germany). Glycerol in plasma (5 μL) and in the interstitial fluid was analyzed with a radiometric kit (Randox Laboratories, GY 105, Montpellier, France). FFA in plasma (5 μL) were estimated colorimetrically with a commercial kit (Randox Laboratories, FA 115, Montpellier, France). All assays were run twice in duplicates.

### Statistical Analysis

Data are presented as means ± S.E.M. Statistical analysis included paired and unpaired tests, both using the two-way ANOVA method with Bonferroni *post hoc* analysis. All statistical comparisons were carried out based on the General Linear Model consisting of subject factor, inter-subject factor Status, within-subject factors OLB and Time, and the interactions Status × OLB and Status × Time. Correlations between measurable factors were explored using Spearman's rank correlation coefficient. The original data were transmutated by power transformations to attain Gaussian data distribution and constant variance in the data and residuals. The homogeneity of the data after power transformations was tested by residual analysis. The difference between medians (Mann-Whitney and Wilcoxon Signed-Rank tests) was applied to compare basal values with exercise-simulated values alone or together with OLB administration. Statistical significance was set at the *P* < 0.05.

## Results

### Hormonal, Bioanalytical, Hemodynamic, and Anthropometric Parameters

**Figure 3:**
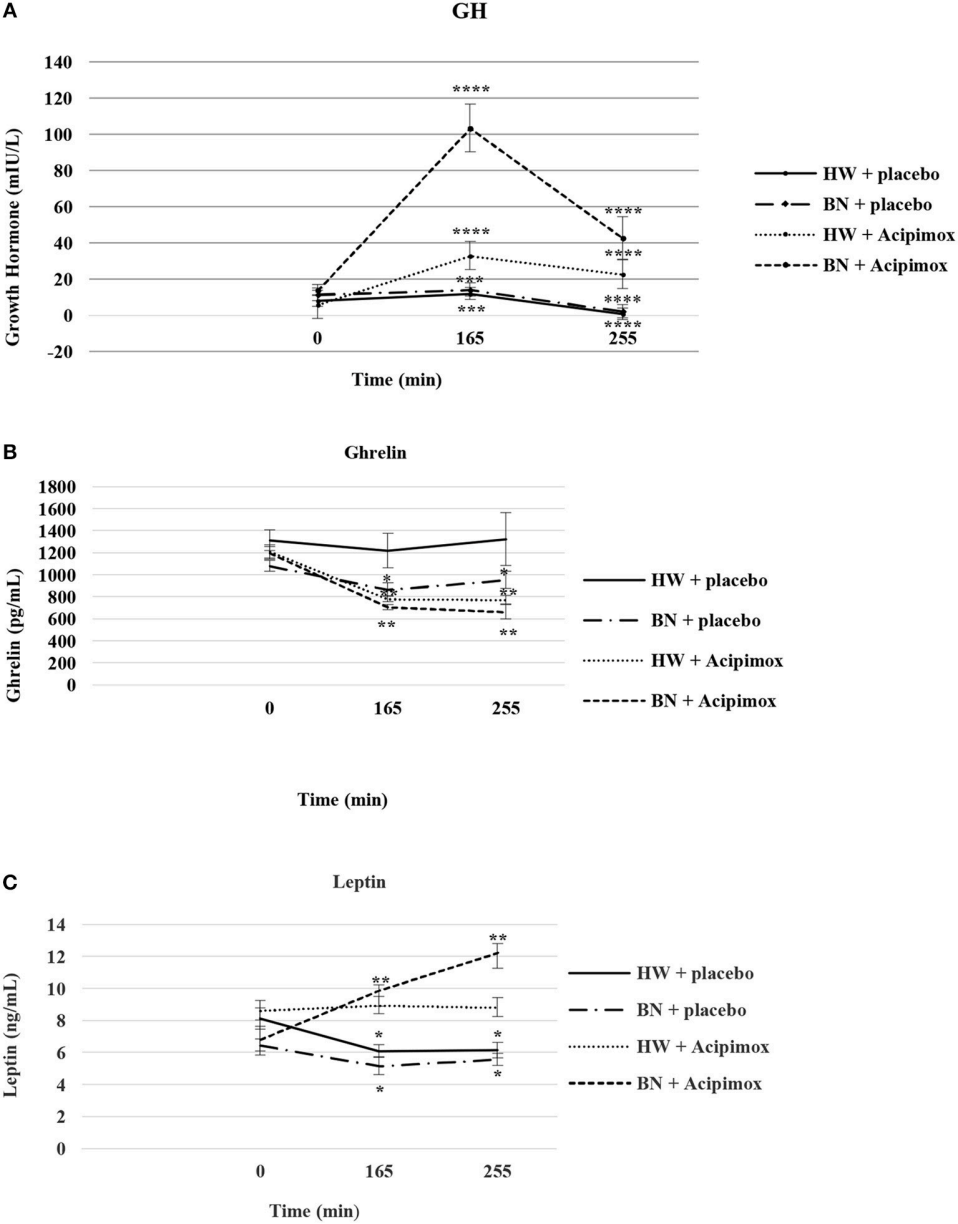
Effect of Acipimox or placebo administration together with exercise on plasma growth hormone **(A)**, ghrelin **(B)**, and leptin **(C)** levels. Values are means ± S.E.M. HW = healthy-weight Czech women, BN = Czech women with bulimia nervosa, *n* = the numbers of subjects are in brackets. Effect of 45 min exercise (at 165 min after the start of the experiment, 2 W/kg of lean body mass [LBM]) and effect of 90 min post-exercise recovery phase (at 255 min after the start of the experiment) alone or together with Acipimox (OLB) administration on plasma growth hormone (GH), ghrelin and leptin levels in healthy-weight Czech women (HW) (*n* = 12) and in Czech women with bulimia nervosa (BN) (*n* = 12). *P < 0.05, **P < 0.01, ***P < 0.001, ****P < 0.0001 vs. resting (basal) values.

**Figure 4:**
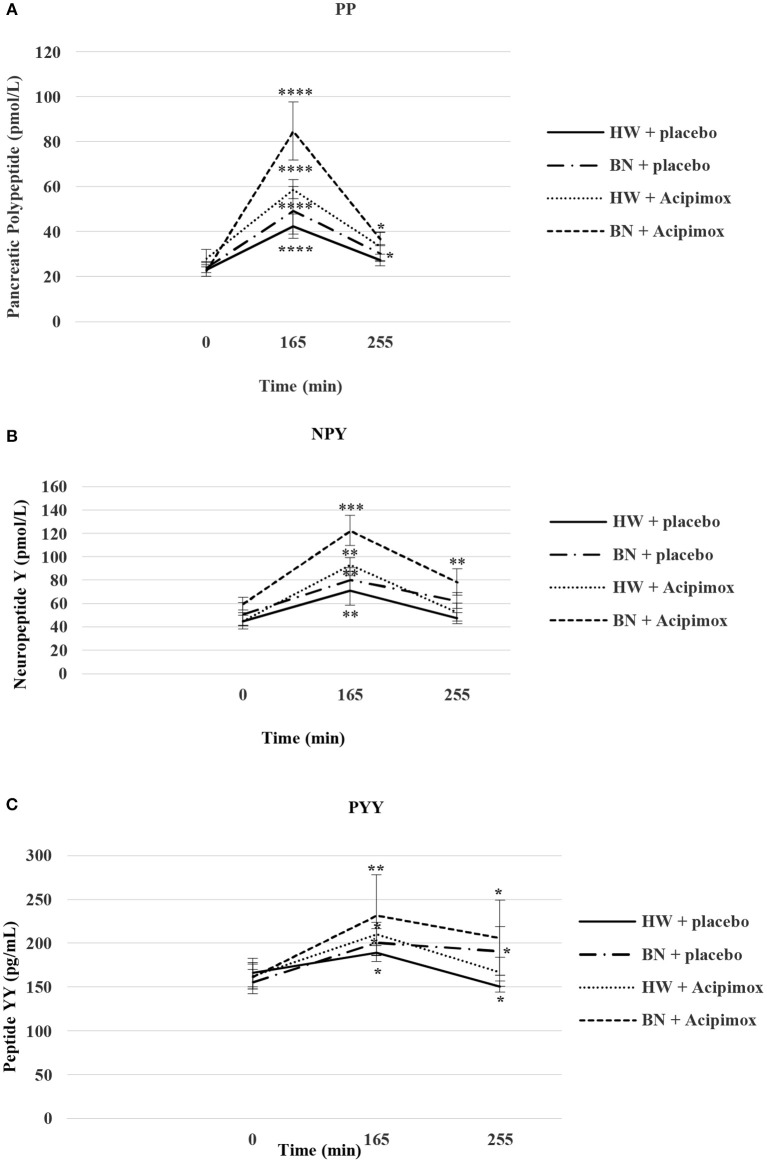
Effect of Acipimox or placebo administration together with exercise on plasma pancreatic polypeptide **(A)**, neuropeptide Y **(B)**, and peptide YY **(C)** levels. Values are means ± S.E.M. HW = healthy-weight Czech women, BN = Czech women with bulimia nervosa, *n* = the numbers of subjects are in brackets. Effect of 45 min exercise (at 165 min after the start of the experiment, 2 W/kg of lean body mass [LBM]) and effect of 90 min post-exercise recovery phase (at 255 min after the start of the experiment) alone or together with Acipimox (OLB) administration on plasma pancreatic polypeptide (PP), neuropeptide tyrosine (NPY), and peptide tyrosine tyrosine (PYY) levels in healthy-weight Czech women (HW) (*n* = 12) and in Czech women with bulimia nervosa (BN) (*n* = 12). *P < 0.05, **P < 0.01, ***P < 0.001, ****P < 0.0001 vs. resting (basal) values.

**Figure 5:**
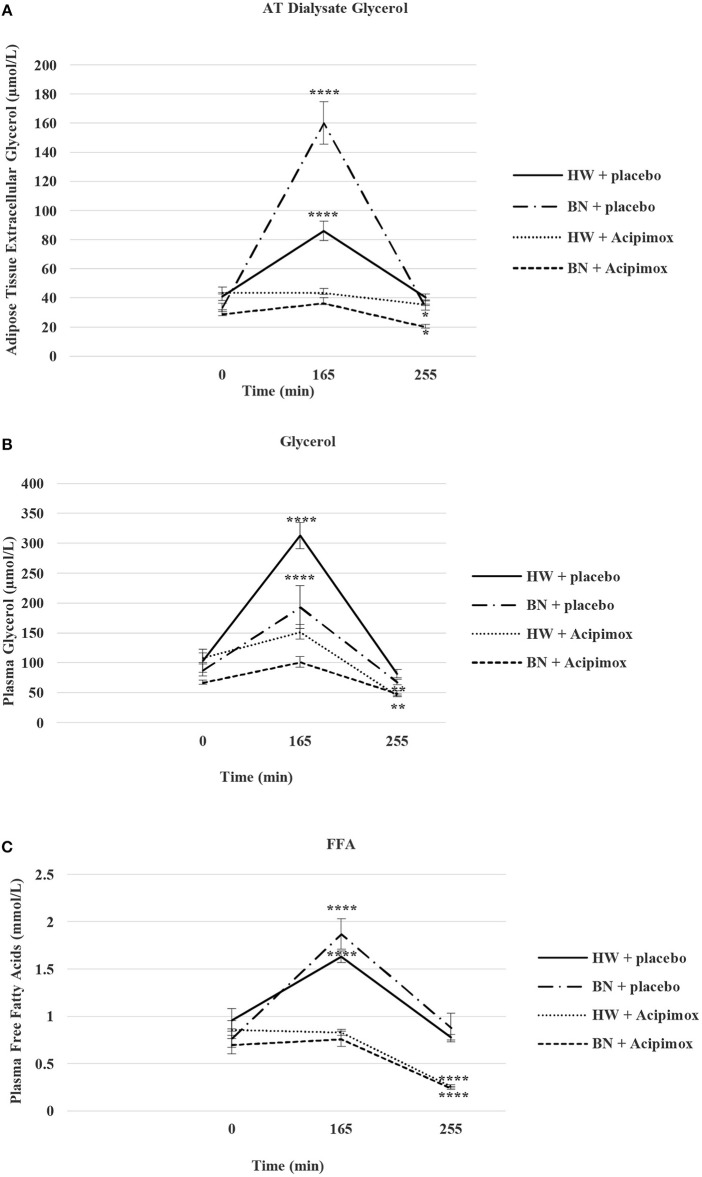
Effect of Acipimox or placebo administration together with exercise on adipose tissue (AT) glycerol **(A)**, and plasma glycerol **(B)**, and plasma free fatty acids **(C)** levels. Values are means ± S.E.M. HW = healthy-weight Czech women, BN = Czech women with bulimia nervosa, *n* = the numbers of subjects are in brackets. Effect of 45 min exercise (at 165 min after the start of the experiment, 2 W/kg of lean body mass [LBM]) and effect of 90 min post-exercise recovery phase (at 255 min after the start of the experiment) alone or together with Acipimox (OLB) administration on adipose tissue (AT) glycerol levels and plasma glycerol and plasma free fatty acids (FFA) levels in healthy-weight Czech women (HW) (*n* = 12) and in Czech women with bulimia nervosa (BN) (*n* = 12). *P < 0.05, **P < 0.01, ****P < 0.0001 vs. resting (basal) values.

Baseline assessments as well as the anthropometric examination and bioimpedance measurement of the study subjects and circulatory responses during rest and exercise alone or together with OLB are shown in Table 1. Baseline plasma and interstitial fluid levels and their exercise-stimulated changes in the study subjects after OLB or placebo treatment are summarized in Tables 2, 3, Figures 3–5, respectively.

**Table 2:** Effect of exercise alone or together with Acipimox administration on plasma adipose tissue-gut-brain axis peptides.

	**0 min**	**45 min**	**45 min**	**90 min**	**90 min**
	**Baseline**	**Exercise (at 165 min of the experiment)**	**Exercise (at 165 min of the experiment)**	**Post-exercise (at 255 min of the experiment)**	**Post-exercise (at 255 min of the experiment)**
		**+placebo 500 mg p.o**.	**+OLB 500 mg p.o**.	**+placebo 500 mg p.o**.	**+OLB 500 mg p.o**.
**GH (mIU/L)**					
HW group (*n =* 12)	7.58 ± 1.16	11.9 ± 1.5^***^	32.9 ± 5.14^****+^	0.73 ± 0.15^****^	22.7 ± 5.4^****#^
BN group (*n =* 12)	11.7 ± 2.35^$^	14.1 ± 3.4^***^	103.4 ± 13.2^****$$++^	2.15 ± 0.4^****$^	42.6 ± 11.7^****$#^
**GHRELIN (pg/mL)**					
HW group (*n =* 12)	1262.4 ± 76.2	1218.3 ± 154.6	779.9 ± 25.7^**^	1322.2 ± 240.5	773.2 ± 37.2^**^
BN group (*n =* 12)	1139.2 ± 53.3	866.5 ± 57.9^*^^$^	706.3 ± 24.8^**^^$^	952.3 ± 77.7^*^^$^	663.6 ± 63.1^**^
**PP (pmol/L)**					
HW group (*n =* 12)	25.63 ± 3.5	42.41 ± 5.51^****^	58.85 ± 4.28^****^^+^	27.38 ± 2.53	33.5 ± 6.39^#^
BN group (*n =* 12)	20.3 ± 2.4^$^	49.41 ± 10.62^****^	84.81 ± 12.89^****$+^	30.39 ± 3.72^*^	36.68 ± 2.97^*^^#^
**NPY (pmol/L)**					
HW group (*n =* 12)	44.2 ± 5.7	71.2 ± 12.6^**^	93.3 ± 5.9^**^	47.4 ± 4.6	52.6 ± 7.7
BN group (*n =* 12)	55.4 ± 7.6^$^	80.5 ± 9.9^**^^$^	122.4 ± 12.9^***$+^	62.5 ± 6.7^$^	78.5 ± 11.4^**^^$$^
**PYY (pg/mL)**					
HW group (*n =* 12)	164.7 ± 15.6	189.1 ± 10.1^*^	210.6 ± 13.3^*^	150.5 ± 6.13^*^	167.2 ± 16.6
BN group (*n =* 12)	158.9 ± 14.1	201 ± 15.4^*^	232 ± 45.8^**^	191.18 ± 27.8^*^	206.1 ± 42.9^*^
**LEPTIN (ng/mL)**					
HW group (*n =* 12)	8.37 ± 0.63	6.09 ± 0.39^*^	8.95 ± 0.53^+^	6.15 ± 0.49^*^	8.83 ± 0.59^#^
BN group (*n =* 12)	6.63 ± 0.51^$^	5.16 ± 0.56^*^	9.88 ± 0.34^**$+^	5.57 ± 0.38^*^	12.25 ± 0.56^**^^#^

*Values are means ± S.E.M. HW = healthy-weight Czech women, BN = Czech women with bulimia nervosa, n = the numbers of subjects are in brackets*.

* P < 0.05,

** P < 0.01,

*** P < 0.001,

**** P < 0.0001 vs. resting (basal) values.

$ P **<** 0.05 BN vs. HW.

$$ P **<** 0.01 BN vs. HW.

+ P **<** 0.05 exercise together with OLB administration vs. exercise alone, 45min.

++ P < 0.01 exercise together with OLB administration vs. exercise alone, 45min.

# P < 0.05 post-exercise recovering phase together with OLB administration vs. post-exercise recovering phase alone, 90 min.

*Effect of 45 min exercise (at 165 min after the start of the experiment, 2 W/kg of lean body mass [LBM]) and effect of 90-min post-exercise recovery phase (at 255 min after the start of the experiment) alone or together with Acipimox (OLB) administration on plasma adipose tissue (AT)-gut-brain axis peptides in healthy-weight Czech women (HW) (n = 12) and in Czech women with bulimia nervosa (BN) (n = 12)*.

**Table 3:** Effect of exercise alone or together with Acipimox administration on adipose tissue and plasma metabolites.

	**0 min**	**45 min**	**45 min**	**90 min**	**90 min**
	**Baseline**	**Exercise (at 165 min of the experiment)**	**Exercise (at 165 min of the experiment)**	**Post-exercise (at 255 min of the experiment)**	**Post-exercise (at 255 min of the experiment)**
		**+placebo 500 mg p.o**.	**+OLB 500 mg p.o**.	**+placebo 500 mg p.o**.	**+OLB 500 mg p.o**.
**AT DIALYSATE EXTRACELLULAR GLYCEROL (μmol/L)**					
HW group (*n =* 12)	42.38 ± 3.2	85.92 ± 6.62^****^	43.5 ± 2.93^++^	40.38 ± 2.35	35.65 ± 2.83^*^
BN group (*n =* 12)	31.2 ± 2.85^$^	160.13 ± 14.5^****^^$$^	36.5 ± 3.66^++++^	33.65 ± 2.17	20.17 ± 1.6^*^^$#^
**PLASMA GLYCEROL (μmol/L)**					
HW group (*n =* 12)	105.81 ± 14	313.2 ± 22^****^	151.7 ± 12.3^++^	81.7 ± 6.6	45.4 ± 2.5^**#^
BN group (*n =* 12)	77.5 ± 7^$^	193.2 ± 36^****$^	101.3 ± 9.1^$+++^	67.8 ± 4.3	48.8 ± 4.4^**#^
**PLASMA FFA (mmol/L)**					
HW group (*n =* 12)	0.91 ± 0.11	1.63 ± 0.06^****^	0.83 ± 0.03^+^	0.78 ± 0.03	0.26 ± 0.017^****#^
BN group (*n =* 12)	0.74 ± 0.08^$^	1.87 ± 0.16^****$^	0.76 ± 0.08^+^	0.88 ± 0.15	0.24 ± 0.015^****#^
**INSULIN (mIU/L)**					
HW group (*n =* 12)	5.21 ± 0.79	4.05 ± 0.26	2.99 ± 0.42^*+^	2.51 ± 0.19^*^	2.48 ± 0.28^*^
BN group (*n =* 12)	2.38 ± 0.33^$$^	2.12 ± 0.37^$$^	1.78 ± 0.33^*^	1.71 ± 0.24^*^	0.76 ± 0.23^**$#^
**GLUCOSE (mmol/L)**					
HW group (*n =* 12)	4.69 ± 0.09	4.45 ± 0.19	4.79 ± 0.11	4.61 ± 0.13	4.36 ± 0.14
BN group (*n =* 12)	4.2 ± 0.12	4.41 ± 0.24	5.6 ± 0.20^**+^	3.72 ± 0.19	4.62 ± 0.18

*Values are means ± S.E.M. HW, healthy-weight Czech women; BN, Czech women with bulimia nervosa, n = the numbers of subjects are in brackets*.

* P < 0.05,

** P < 0.01,

**** P < 0.0001 vs. resting (basal) values.

$ P < 0.05 BN vs. HW.

$$ P < 0.01 BN vs. HW.

+ P < 0.05 exercise together with OLB administration vs. exercise alone, 45min.

++ P < 0.01 exercise together with OLB administration vs. exercise alone, 45 min.

+++ P < 0.001 exercise together with OLB administration vs. exercise alone, 45 min.

++++ P < 0.0001 exercise together with OLB administration vs. exercise alone, 45 min.

# P < 0.05 post-exercise recovering phase together with OLB administration vs. post-exercise recovering phase alone, 90 min.

*Effect of 45 min exercise (at 165 min after the start of the experiment, 2 W/kg of lean body mass [LBM]) and effect of 90 min post-exercise recovery phase (at 255 min after the start of the experiment) alone or together with Acipimox (OLB) administration on adipose tissue (AT) glycerol and plasma glycerol levels, plasma free fatty acids (FFA), blood glucose, and plasma insulin levels in healthy-weight Czech women (HW) (n = 12), and in Czech women with bulimia nervosa (BN) (n = 12)*.

### BMI and %BF

BMI and % BF were similar in BN and HW under basal condition over a total of 2 weeks (BMI [week + placebo]: 21.85 ± 0.60 vs. 20.76 ± 0.88 kg/m^2^ in HW, not significant [NS]; BMI [week + OLB]: 22.36 ± 0.5 vs. 21.6 ± 0.75 kg/m^2^ in HW, NS; % BF [week + placebo]: 22.7 ± 0.75 vs. 21.5 ± 2.46 in HW, NS; % BF [week + OLB]: 23.1 ± 0.8 vs. 22.4 ± 2.6 in HW, NS, respectively) (Table 1).

### Circulatory Responses Under Rest and During an Acute Bout of Exercise Together With Orally Administered OLB or Placebo

The resting heart rate was lower in BN compared with HW (55 ± 4.5 vs. 72 ± 2.5 beats/min in HW, *P* < 0.05). The resting systolic and diastolic blood pressure were decreased in BN compared with HW (systolic blood pressure: 90 ± 3.5 vs. 121 ± 4.7 mmHg in HW, *P* < 0.05; diastolic blood pressure: 54 ± 3.5 vs. 72 ± 2.7 mmHg in HW, *P* < 0.05, respectively). Exercise stimulation together with orally administered placebo or OLB led to significant increases in heart rate, systolic and diastolic blood pressure in both groups when compared with resting values (Table 1).

### Basal and Exercise-Stimulated Plasma GH Levels Together With Orally Administered OLB or Placebo

Average basal plasma GH levels were significantly increased in BN compared with HW (11.7 ± 2.35 vs. 7.58 ± 1.16 mIU/L in HW, *P* < 0.05). Plasma GH levels rose significantly after the acute bout of exercise together with orally administered placebo in BN and HW (14.1 ± 3.4 vs. 11.9 ± 1.5 mIU/L in HW, *P* < 0.001). In the subsequent week, orally administered OLB 1 h before the start of exercise bout resulted in plasma GH levels increase in BN and HW, to a greater extent in BN (103.4 ± 13.2 vs. 32.9 ± 5.14 mIU/L in HW, *P* < 0.0001). Plasma GH concentrations decreased significantly in HW after the recovering phase together with orally administered placebo compared with BN (2.15 ± 0.4 vs. 0.73 ± 0.15 mIU/L in HW, *P* < 0.0001). Vice versa, plasma GH concentrations were significantly increased after the recovering phase together with orally administered OLB in BN and HW (42.6 ± 11.7 vs. 22.7 ± 5.4 mIU/L in HW, *P* < 0.0001) (Table 2, Figure 3). Immediate exercise-induced plasma GH levels were increased in both groups with placebo, but more in BN with OLB.

### Basal and Exercise-Stimulated Plasma Ghrelin Levels Together With Orally Administered OLB or Placebo

Average basal plasma ghrelin levels were comparable in BN and HW (1139.2 ± 53.3 vs. 1262.4 ± 76.2 pg/mL in HW). In BN patients plasma ghrelin levels significantly decreased after 45 min exercise with placebo compared to HW (866.5 ± 57.9 vs. 1218.3 ± 154.6 pg/mL in HW, *P* < 0.05). In BN and HW plasma ghrelin concentrations decreased after the acute bout of exercise together with orally administered OLB (706.3 ± 24.8 vs. 779.9 ± 25.7 pg/mL in HW, *P* < 0.01). In BN plasma ghrelin concentrations remained significantly lower after the recovering phase together with orally administered placebo in comparison with HW (952.3 ± 77.7 vs. 1322.2 ± 240.5 pg/mL in HW, *P* < 0.05). In both groups plasma ghrelin concentrations were still significantly decreased after the recovering phase together with orally administered OLB (663.6 ± 63.1 vs. 773.2 ± 37.2 pg/mL in HW, *P* < 0.01) (Table 2, Figure 3). Immediate exercise-induced plasma ghrelin levels were decreased only in BN with placebo, but with OLB they were decreased in both groups.

### Basal and Exercise-Stimulated Plasma PP Levels Together With Orally Administered OLB or Placebo

Average basal plasma PP concentrations were significantly lower in BN when compared with HW (20.3 ± 2.4 vs. 25.63 ± 3.5 pmol/L in HW, *P* < 0.05). Plasma PP levels rose significantly after the acute bout of exercise together with orally administered placebo in BN and HW (49.41 ± 10.62 vs. 42.41 ± 5.51 pmol/L in HW, *P* < 0.0001). In the subsequent week orally administered OLB 1 h before the start of the exercise bout resulted in an increase of plasma PP levels in BN and HW, to a greater extent in BN (84.81 ± 12.89 vs. 58.85 ± 4.28 pmol/L in HW, *P* < 0.0001). Plasma PP concentrations decreased more in HW after the recovering phase together with orally administered placebo in comparison with BN (30.39 ± 3.72 vs. 27.38 ± 2.53 pmol/L in HW). Plasma PP concentrations were significantly increased after the recovering phase together with orally administered OLB in BN and HW (36.68 ± 2.97 vs. 33.5 ± 6.39 pmol/L in HW, *P* < 0.05) (Table 2, Figure 4). Immediate exercise-induced plasma PP levels were increased in both groups with placebo, but with OLB they were increased more in BN.

### Basal and Exercise-Stimulated Plasma NPY Levels Together With Orally Administered OLB or Placebo

Average basal plasma NPY concentrations were significantly increased in BN compared with HW (55.4 ± 7.6 vs. 44.2 ± 5.7 pmol/L in HW, *P* < 0.05). Plasma NPY concentrations rose significantly after the acute bout of exercise together with orally administered placebo in BN and HW (80.5 ± 9.9 vs. 71.2 ± 12.6 pmol/L in HW, *P* < 0.01). In the subsequent week orally administered OLB 1 h before the start of the exercise bout resulted in an increase of even elevated NPY levels exclusively in BN (122.4 ± 12.9 vs. 93.3 ± 5.9 pmol/L in HW, *P* < 0.001). Plasma NPY concentrations returned to basal values after the recovering phase together with orally administered placebo in BN and HW (62.5 ± 6.7 vs. 47.4 ± 4.6 pmol/L in HW). Plasma NPY concentrations were above basal values after the recovering phase together with orally administered OLB in BN (78.5 ± 11.4 vs. 52.6 ± 7.7 pmol/L in HW, *P* < 0.01) (Table 2, Figure 4). Immediate exercise-induced plasma NPY levels were increased in both groups with placebo, but with OLB they were increased more in BN.

### Basal and Exercise-Stimulated Plasma PYY Levels Together With Orally Administered OLB or Placebo

Average basal plasma PYY levels were comparable in BN and HW (158.9 ± 14.1 vs. 164.7 ± 15.6 pg/mL in HW). Plasma PYY levels rose significantly after the acute bout of exercise together with orally administered placebo in BN and HW (201 ± 15.4 vs. 189.1 ± 10.1 pg/mL in HW, *P* < 0.05). In the subsequent week orally administered OLB 1 h before the start of exercise bout resulted in an increase of plasma PYY levels in BN and HW, to a greater extent in BN (232 ± 45.8 vs. 210.6 ± 13.3 pg/mL in HW, *P* < 0.01). Plasma PYY levels increased significantly more in BN after the recovering phase together with orally administered placebo compared with HW (191.18 ± 27.8 vs. 150.5 ± 6.13 pg/mL in HW, *P* < 0.05). Plasma PYY concentrations were significantly increased after the recovering phase together with orally administered OLB exclusively in BN when compared to baseline values (206.1 ± 42.9 vs. 158.9 ± 14.1 pg/mL in BN, *P* < 0.05) (Table 2, Figure 4). Immediate exercise-induced plasma PYY levels were increased in both groups with placebo, but with OLB they were increased more in BN.

### Basal and Exercise-Stimulated Plasma Leptin Levels Together With Orally Administered OLB or Placebo

Average basal plasma leptin concentrations were significantly lower in BN compared with HW (6.63 ± 0.51 vs. 8.37 ± 0.63 ng/mL in HW, *P* < 0.05). Plasma leptin concentrations decreased significantly after the acute bout of exercise together with orally administered placebo in BN and HW (5.16 ± 0.56 vs. 6.09 ± 0.39 ng/mL in HW, *P* < 0.05). Plasma leptin levels increased significantly after the acute bout of exercise together with orally administered OLB in BN in comparison with HW (9.88 ± 0.34 vs. 8.95 ± 0.53 ng/mL in HW, *P* < 0.01). Plasma leptin levels decreased significantly after the recovering phase together with orally administered placebo in BN and HW (5.57 ± 0.38 vs. 6.15 ± 0.49 ng/mL in HW, *P* < 0.05). Plasma leptin concentrations rose significantly after the recovering phase together with orally administered OLB more in BN in comparison with HW (12.25 ± 0.56 vs. 8.83 ± 0.59 ng/mL in HW, *P* < 0.01) (Table 2, Figure 3). Immediate exercise-induced plasma leptin levels were decreased in both groups with placebo, but with OLB they were increased more in BN.

### Basal and Exercise-Stimulated AT Dialysate Extracellular Glycerol Levels Together With Orally Administered OLB or Placebo

Average basal AT dialysate extracellular glycerol concentrations were significantly lower in BN compared with HW (31.2 ± 2.85 vs. 42.38 ± 3.2 μmol/L in HW, *P* < 0.05). AT dialysate extracellular glycerol levels rose significantly after the acute bout of exercise together with orally administered placebo to a greater extent in BN (160.13 ± 14.5 vs. 85.92 ± 6.62 μmol/L in HW, *P* < 0.0001). AT dialysate extracellular glycerol concentrations decreased significantly after the acute bout of exercise together with orally administered OLB, to a greater extent in BN (36.5 ± 3.66 vs. 43.5 ± 2.93 μmol/L in HW, *P* < 0.0001). AT dialysate extracellular glycerol levels returned to the basal values after the recovering phase together with orally administered placebo in BN and HW (33.65 ± 2.17 vs. 40.38 ± 2.35 μmol/L in HW). AT dialysate extracellular glycerol concentrations decreased significantly below the basal concentrations after the recovering phase together with orally administered OLB, to a greater extent in BN (20.17 ± 1.6 vs. 35.65 ± 2.83 μmol/L in HW, *P* < 0.05) (Table 3, Figure 5). Immediate exercise-induced AT extracellular glycerol concentrations were increased more in BN with placebo, but with OLB they were decreased more in BN.

### Basal and Exercise-Stimulated Plasma Glycerol Levels Together With Orally Administered OLB or Placebo

Average basal plasma glycerol concentrations were significantly lower in BN compared with HW (77.5 ± 7 vs. 105.81 ± 14 μmol/L in HW, *P* < 0.05). Plasma glycerol concentrations rose significantly after the acute bout of exercise together with orally administered placebo in BN and HW (193.2 ± 36 vs. 313.2 ± 22 μmol/L in HW, *P* < 0.0001). Plasma glycerol levels decreased significantly after the acute bout of exercise together with orally administered OLB, to a greater extent in BN in comparison with the acute bout of exercise together with orally administered placebo (101.3 ± 9.1 vs. 151.7 ± 12.3 μmol/L in HW, *P* < 0.001). Plasma glycerol levels returned to the basal values after the recovering phase together with orally administered placebo in BN and HW (67.8 ± 4.3 vs. 81.7 ± 6.6 μmol/L in HW). Plasma glycerol levels significantly decreased below the basal values after the recovering phase together with orally administered OLB in BN and HW (48.8 ± 4.4 vs. 45.4 ± 2.5 μmol/L in HW, *P* < 0.01) (Table 3, Figure 5). Immediate exercise-induced plasma glycerol levels were increased in both groups with placebo, but with OLB they were decreased more in BN.

### Basal and Exercise-Stimulated Plasma FFA Levels Together With Orally Administered OLB or Placebo

Average basal plasma FFA levels were significantly decreased in BN in comparison with HW (0.74 ± 0.08 vs. 0.91 ± 0.11 mmol/L in HW, *P* < 0.05). Plasma FFA levels rose significantly after the acute bout of exercise together with orally administered placebo in BN and HW (1.87 ± 0.16 vs. 1.63 ± 0.06 mmol/L in HW, *P* < 0.0001). Plasma FFA levels decreased significantly to the basal values after the acute bout of exercise together with orally administered OLB in BN and HW in comparison with the acute bout of exercise together with orally administered placebo (0.76 ± 0.08 vs. 0.83 ± 0.03 mmol/L in HW, *P* < 0.05). Plasma FFA levels returned to the basal levels after the recovering phase together with orally administered placebo in BN and HW (0.88 ± 0.15 vs. 0.78 ± 0.03 mmol/L in HW). Plasma FFA levels decreased significantly below the basal levels after the recovering phase together with orally administered OLB in BN and HW (0.24 ± 0.015 vs. 0.26 ± 0.017 mmol/L in HW, *P* < 0.0001) (Table 3, Figure 5). Immediate exercise-induced plasma FFA levels were increased in both groups with placebo, but with OLB they were decreased in both groups.

### Basal and Exercise-Stimulated Plasma Insulin Levels Together With Orally Administered OLB or Placebo

Average basal plasma insulin levels were significantly lower in BN patients compared with HW (2.38 ± 0.33 vs. 5.21 ± 0.79 mIU/L in HW, *P* < 0.01). Plasma insulin levels approached the basal values after the acute bout of exercise together with orally administered placebo in BN and HW (2.12 ± 0.37 vs. 4.05 ± 0.26 mIU/L in HW). Plasma insulin levels decreased significantly after the acute bout of exercise together with orally administered OLB in both groups (1.78 ± 0.33 vs. 2.99 ± 0.42 mIU/L in HW, *P* < 0.05). Plasma insulin levels decreased significantly after post-exercise recovering phase together with orally administered placebo in both groups (1.71 ± 0.24 vs. 2.51 ± 0.19 mIU/L in HW, *P* < 0.05), and decreased significantly even more after post-exercise recovering phase with orally administered OLB in BN patients when compared to baseline values (0.76 ± 0.23 vs. 2.38 ± 0.33 mIU/L in BN, *P* < 0.01) (Table 3). Immediate exercise-induced plasma insulin levels approached the basal values in both groups with placebo, but with OLB they were decreased in both groups.

### Basal and Exercise-Stimulated Plasma Blood Glucose Levels Together With Orally Administered OLB or Placebo

Average basal plasma blood glucose levels were similar in BN and HW (4.2 ± 0.12 vs. 4.69 ± 0.09 mmol/L in HW). Plasma blood glucose levels were over the basal values in BN after the acute bout of exercise together with orally administered placebo (4.41 ± 0.24 vs. 4.45 ± 0.19 mmol/L in HW). They increased significantly after the acute bout of exercise together with orally administered OLB in BN when compared to baseline values (5.6 ± 0.20 vs. 4.2 ± 0.12 mmol/L in BN, *P* < 0.01). Plasma blood glucose levels were under the basal values after the recovering phase together with orally administered placebo in both groups (3.72 ± 0.19 vs. 4.61 ± 0.13 mmol/L in HW), but they were over the basal values after the recovering phase together with orally administered OLB in BN (4.62 ± 0.18 vs. 4.36 ± 0.14 mmol/L in HW) (Table 3). Immediate exercise-induced plasma blood glucose levels were over the basal values in BN with placebo, but with OLB they were increased more in BN.

### Correlations

Basal plasma glucose levels negatively correlated with plasma FFA levels in BN (*r* = −0.56, *P* = 0.01). Basal plasma glycerol levels positively correlated with plasma FFA levels in BN (*r* = 0.72, *P* = 0.0001) and in HW (*r* = 0.81, *P* = 0.0001). Plasma GH levels positively correlated with plasma NPY levels after the acute bout of exercise together with orally administered OLB exclusively in BN (*r* = 0.74, *P* = 0.01). Plasma glycerol levels positively correlated with plasma FFA levels after the acute bout of exercise together with orally administered OLB in BN (*r* = 0.81, *P* = 0.001) and in HW (*r* = 0.73, *P* = 0.001). Plasma insulin concentrations positively correlated with plasma leptin concentrations after the acute bout of exercise together with orally administered OLB in HW (*r* = 0.71, *P* = 0.05).

## Discussion

In the present placebo-controlled study, basal GH and NPY plasma concentrations were increased and baseline fasting PP and leptin plasma levels were decreased, but not baseline fasting ghrelin and PYY levels in BN. GH, PP, and PYY plasma levels were comparably elevated after short-term exercise with placebo administration in both groups, but acute exercise caused suppression of plasma ghrelin concentrations exclusively in BN. The anti-hyperlipidemic drug OLB administered orally 1 h before the start of short-term exercise caused a significant elevation of plasma GH, PP, and PYY levels in BN and HW and a significant suppression of plasma ghrelin in both groups (Table 2, Figures 3, 4).

Some studies have reported a decrease (9, 35) or an increase (36) in plasma ghrelin levels following exercise, whereas others have not observed any changes (37). These results may depend on several factors such as duration or intensity of exercise, body composition, nutritional status as well as the timing of food intake. Indeed, the stomach is heavily innervated by the SNS and both *in vitro* and *in vivo* studies have shown secretion of ghrelin in response to physical stress and sympathetic activation signaled *via* the beta1-adrenergic receptor present on the gastric ghrelin cells and that NE and E act as direct ghrelin secretagogues (38–41). As ghrelin is an endogenous ligand of the GH secretagogue receptor, it may be supposed that circulating ghrelin levels would be linked to endogenous GH release (Figure 1). Thus, there could be an inhibitory feedback such that reduction of ghrelin occurs even when plasma GH levels are elevated (42, 43). However, in our study, we did not find any positive feedback loop between ghrelin and GH and any contribution of ghrelin to the exercise-stimulated increase in GH.

The shift in hypothalamic NPY and its a mutual relationship with ghrelin and leptin plasma concentrations and also the mechanisms that underlie their reciprocal actions have not yet been described. Recently, Zhang et al. (44) showed in the course of development of the rat model of type 2 diabetes mellitus that hypothalamic NPY mRNA expression capacity was negatively correlated with plasma ghrelin and positively correlated with plasma leptin. Moreover, GH increases plasma leptin levels (45) and leptin is a potent inhibitor of ghrelin (46, 47) which can inhibit gastric ghrelin release. Furthermore, it was shown that the rise of plasma PP and PYY levels may be involved in the suppression of release of ghrelin by inhibiting the synthesis of ghrelin (48–50). Resistance exercise has been reported to elevate GH levels to a greater extent compared to aerobic exercise (51), which may clarify why resistance exercise in comparison with aerobic exercise has a considerable inhibitory influence on plasma ghrelin levels. In our study, it is likely that a lesser rise in plasma GH, PP, and PYY levels induced by moderate intensity exercise did not invoke GH co-feedback inhibition of ghrelin secretion in HW (Table 2, Figures 3, 4). Conversely, in BN with increased baseline fasting GH levels and unchanged baseline plasma ghrelin levels, a further increase of plasma GH, PP, and PYY levels induced by exercise alone invoked GH co-feedback inhibition of plasma ghrelin levels. Therefore, it can be suggested that in BN, unlike HW, plasma ghrelin levels best mirror nutritional status rather than specific patterns of disordered eating behavior (5).

In contrast to exercise alone, the anti-hyperlipidemic drug OLB administered orally 1 h before the start of short-term exercise caused not only a significant rise in plasma GH, PP, PYY, and leptin levels but also a decrease in plasma ghrelin levels in both groups (Table 2, Figures 3, 4). Thus, it is possible that only high absolute plasma GH, PP, and PYY levels after exercise together with OLB administration result in an important lowering of plasma ghrelin levels as well in HW. Another possibility is that a decrease in plasma ghrelin mirrors alterations in plasma FFA and glycerol levels during exercise in combination with OLB administered orally in BN and HW (Table 3, Figure 5). Ghrelin is a food initiating signal and in accordance with this opinion the lowering of lipolysis in AT would lower the secretion of ghrelin because the mobilization of FFA increases during exercise and starvation, and is blocked by caloric intake (42). Interestingly, FFA have been reported (52, 53) to reduce ghrelin concentrations independently of GH concentrations in humans. However, we observed lowered plasma ghrelin concentrations promptly after short-term exercise in BN, whereas plasma FFA and glycerol concentrations raised to the same extent as in HW in whom plasma ghrelin was not affected. Furthermore, the drop of plasma ghrelin concentrations was found after short-term exercise together with OLB administered orally in both BN and HW, when FFA concentrations restored to baseline, or in the recovering phase when FFA levels were infraphysiologically suppressed and GH, PP, PYY, and leptin levels were still higher than basal levels in both groups. These observations lead us to suggest that elevated GH, PP, PYY, and leptin concentrations stimulated by short-term exercise together with OLB administration may not be immediately signaled through FFA and may not affect ghrelin release in both BN and HW. This supports the view suggesting a FFA-independent mechanism of OLB.

Additionally, administration of OLB together with exercise stopped the decline in plasma leptin concentrations in both BN and HW. In fact, plasma leptin concentrations were significantly elevated to a greater extent in BN after the post-exercise recovering phase together with OLB administration (Table 2, Figure 3). Furthermore, post-exercise plasma GH levels together with OLB administration correlated positively with NPY in BN and these findings support our suggestion that exercise-stimulated GH secretion is up-regulated by NPY (10) and that the PP, PYY, and leptin may exert a co-feedback action with GH on the suppression of ghrelin secretion (Table 2, Figures 3, 4). It was shown that PP and PYY efficiently decrease plasma ghrelin concentrations in humans and animals (48–50).

We found significantly higher AT dialysate extracellular glycerol levels during short-term exercise in BN (Table 3, Figure 5). Conversely, administration of OLB during short-term exercise led to a substantial decrease of AT dialysate extracellular glycerol levels and therefore to a greater reduction of lipolysis in BN. It was shown that nicotinic acid is capable of inhibiting isoproterenol-stimulated lipolysis in adipocytes (54). Thus, OLB completely inhibited and overruled the effect of exercise-stimulated lipolysis *via* beta-adrenergic pathway of cAMP production on AT dialysate extracellular glycerol levels, leading to a higher reduction of lipolysis in sc abdominal AT of BN. NE in AT is one of the most potent stimulators of glycerol and FFA release, and thus, our results demonstrate that OLB has a beta-sympathicolytic effect in sc abdominal AT as demonstrated previously for nicotinic acid (21, 55) while blocking the NE effect on FFA release. We suggest that OLB acts *via* its inhibition of cAMP production, rather than *via* alternative cAMP-independent pathways (28, 29, 56). Furthermore, BN patients are extremely sensitive to OLB, and abnormal local modification of both adrenergic and NPY-PYY-PP-ergic influences *via* their inhibition of cAMP production in sc abdominal AT may abolish lipolysis during short-term exercise to a greater extent in BN. Moreover, GH increases lipolysis which favors FFA as a source of energy during physical activity for the hormone-sensitive lipase, indirectly attenuating the capability of adipocytes to respond to anti-lipolytic alpha_2_-adrenergic dependent lowering of cAMP production (57). In the present study, OLB after the 45 min exercise inhibited the effect of supraphysiological GH levels on lipolysis in sc abdominal AT much more in BN. AT dialysate extracellular glycerol is the final breakdown product during baseline and exercise-stimulated lipolysis and AT dialysate extracellular glycerol, once released, does not seem to be re-esterified in sc abdominal AT unlike the released FFA. Thus, plasma glycerol may be considered as a preferable immediate indicator of lipolysis than FFA. In both groups, short-term exercise with placebo induced similar increases in plasma glycerol levels. However, OLB administered orally together with short-term exercise decreased plasma glycerol levels to a greater extent in BN, suggesting an effect of an increased turnover of plasma glycerol reflecting altered metabolic status of BN.

## Conclusion

Taken together, the present data support the hypothesis that an exercise-stimulated rise in GH, PP, and PYY concentrations lowers ghrelin levels exclusively in BN, but do not support the hypothesis that exercise-induced minor increase in GH, PP, and PYY levels inhibits ghrelin secretion in HW. Simultaneously, this study supports the hypothesis that the decrease in ghrelin levels observed after OLB administration during short-term exercise and after the post-exercise recovering phase may be consistent with a potential negative co-feedback of GH, PP, PYY, and leptin on ghrelin secretion in both groups. Therefore, ghrelin may be a potential discriminator between patients with endocrine disturbances in energy imbalance such as in BN and AN, but not in HW. Altogether, our results support the hypothesis that a higher reduction of peripheral lipolysis thanks to the anti-lipolytic drug OLB exists in BN, and that OLB acts *via* its inhibition of cAMP production, rather than *via* alternative cAMP-independent pathways.

Therefore, the results of a randomized, single-blind, microdialysis study should contribute further to the development of an innovative generation of anti-lipolytic drugs as well as the AT-gut-brain axis synthetic analogs that could alter synaptic cleft concentrations of NE and E and therefore lipid mobilization and energy expenditure.

## Author Contributions

KS designed the study and performed clinical and research experiments of the study, interpreted and evaluated of the results, and wrote the manuscript. JN designed the study and supervised the experimental sessions, evaluated of the results, reviewed and edited the manuscript. KV selected and clinically evaluated of the study subjects, and performed clinical experiments of the study. MH researched and analyzed statistical data, researched and evaluated of the results. HP selection and clinical evaluation of eligible patients to establish the diagnosis of bulimia nervosa (psychiatrist). VH selected and clinically evaluated of the study subjects, and drafted the manuscript. All authors approved the final version.

## Conflict of Interest Statement

The authors declare that the research was conducted in the absence of any commercial or financial relationships that could be construed as a potential conflict of interest.

## Abbreviations

BN: Czech women with bulimia nervosa
AN: anorexia nervosa
HW: healthy-weight Czech women
OLB: Acipimox
BMI: body mass index
% BF: body fat content determination
NS: not significant
LBM: lean body mass
AT: adipose tissue
sc: subcutaneous
GH: growth hormone
PP: pancreatic polypeptide
NPY: neuropeptide tyrosine
PYY: peptide tyrosine tyrosine
SNS: sympathetic nervous system
HCA: hydroxy-carboxylic acid
FFA: free fatty acids
NE: norepinephrine
E: epinephrine
ARC: arcuate nucleus.
